# Magnetic resonance imaging presentation of deep infiltrating endometriosis nodules before and after pregnancy: A case series

**DOI:** 10.1371/journal.pone.0223330

**Published:** 2019-10-04

**Authors:** Anne Elodie Millischer, Louis Marcellin, Pietro Santulli, Chloe Maignien, Mathilde Bourdon, Bruno Borghese, François Goffinet, Charles Chapron

**Affiliations:** 1 Centre de Radiologie IMPC Bachaumont Pole femme-mere-enfant, Paris, France; 2 Université Paris Descartes, Sorbonne Paris Cité, Faculté de Médecine, Assistance Publique–Hôpitaux de Paris (AP-HP), Service de Chirurgie Gynécologie Obstétrique II et Médecine de la Reproduction, Hôpital Universitaire Paris Centre (HUPC), Centre Hospitalier Universitaire (CHU) Cochin, Paris, France; 3 Equipe Stress Oxydant, Prolifération Cellulaire et Inflammation, Département Développement, Reproduction, Cancer, Inserm U1016, Université Paris Descartes, Sorbonne Paris Cité, Faculté de Médecine, AP-HP, HUPC, CHU Cochin, Paris, France; 4 Equipe Génomique, Epigénétique et Physiopathologie de la Reproduction, Département Développement, Reproduction, Cancer, Inserm U1016, Université Paris Descartes, Sorbonne Paris Cité, Faculté de Médecine, AP-HP, HUPC, CHU Cochin, Paris, France; 5 Port Royal Maternity Unit, Cochin Hospital, Assistance Publique des Hôpitaux de Paris, DHU Risks and Pregnancy, Paris Descartes University, Paris, France; University at Buffalo, UNITED STATES

## Abstract

**Objective:**

To compare the magnetic resonance imaging (MRI) features of deep infiltrating endometriosis (DIE) lesions before and after pregnancy.

**Design:**

Retrospective study.

**Setting:**

A single French university tertiary referral hospital.

**Patients:**

Twenty-one women without a prior history of surgery for endometriosis with a radiological diagnosis by MRI with two sets of examinations performed before and after pregnancy.

**Interventions:**

The volumes of the lesions were compared using the same protocol before and after pregnancy based on MRI (1.5 T) examinations by a single experienced radiologist who is a referring practitioner for image-based diagnosis of endometriosis.

**Main outcome measure(s):**

The DIE lesion volume.

**Measurements and main results:**

Between October 2012 and December 2016, a total of 21 patients (67 lesions) were included and compared before and after pregnancy. The mean time interval between the MRI before pregnancy and delivery was 19.6 ± 8.5 months (median: 17.6, IQR 13.5–25.2 months). The mean time interval between delivery and the MRI after pregnancy was 11.0 ± 6.4 months (median: 8.3, IQR 6–15.2 months). The mean overall DIE lesion volume by MRI was significantly higher before pregnancy compared to after pregnancy (2,552 ± 3,315 mm^3^ vs. 1,708 ± 3,266 mm^3^, respectively, p < 0.01). The mean volume by MRI of the largest lesion of each patient was significantly higher before pregnancy compared to after pregnancy (4,728 ± 4,776 mm3 vs. 3165 ± 5299 mm^3^; p < 0.01).

**Conclusion:**

Our data indicate a favorable impact of pregnancy on DIE lesion volumes as measured by MRI.

## Introduction

Endometriosis is a common chronic benign hormone-dependent gynecological disorder associated with persistent pelvic pain and/or infertility [1, 2] that is characterized by the development of glandular and stromal endometrium-like tissues at ectopic locations.[3] At present, imaging procedures such as ultrasonography (US),[4] and magnetic resonance imaging (MRI) are suitable for diagnosing and for providing follow-up of endometriotic lesions (endometrioma—OMA and deep infiltrating endometriosis—DIE), and allow for a non-surgical diagnosis of endometriosis,[5, 6] without a need for histological confirmation.[5, 7, 8]

The data available regarding the interrelationships between pregnancy and endometriosis are limited or of poor-quality.[9–11] Historically, pregnancy is typically considered to have a positive -albeit temporary- effect on endometriosis, which is most likely due to anovulation, amenorrhea, and pregnancy-related hormonal changes that prevent bleeding in the ectopic endometriotic tissue.[12] Varying degrees of growth changes of ectopic endometriotic lesions have been observed.[11] In addition, severe but rare and unpredictable complications during pregnancy have been reported with puerperal changes in ectopic lesions.[13]

Preliminary data indicate that pregnancies in conjunction with endometriosis are associated with increased adverse pregnancy outcomes such as spontaneous miscarriage,[14] preterm birth, preeclampsia, small-for-gestational-age offspring, and obstetric hemorrhages.[15–22] One of the suspected etiologies stems from the potential increased risk of defective deep placentation.[23, 24] Moreover, endometriosis-like glands have previously been reported to be in close contact with the fetal membranes in pregnant women who have endometriosis.[25]

During pregnancy, the endometrium undergoes decidualization (initiated during the secretory phase of the menstrual cycle and persisting when implantation of the blastocyst occurs) that is mainly induced by progesterone.[26] This decidualization results in conversion of the endometrium into a specialized uterine tissue,[27] thereby controlling trophoblast invasion and guaranteeing optimal accommodation of the gestation.[28] This transformation of the endometrium involves different metabolic, hormonal, angiogenic, and immune system changes related to steroid exposure.[29] Decidualization also occurs in ectopic endometriotic lesions.[30, 31] It is not clear whether these changes in ectopic lesions persist after delivery. A previous study evaluated the clinical features and the change in size based on US during pregnancy of 25 OMA,[32] and a 3-case series of deep infiltrating endometriosis (DIE) lesions,[30] both of which exhibited a change in size. However, no data are available regarding the progression of DIE lesions before and after pregnancy based on MRI.

Therefore, the aim of this retrospective study was to compare the DIE lesion volume by MRI before and after pregnancy.

## Materials and methods

### Study design

This study was approved by the National Data Protection Authority (Commission Nationale de l’Informatique et des Libertés, CNIL n° 1755849). French regulations stipulate that this study is exempt from Institutional Review Board (IRB) review because it is an observational study using anonymized data from medical records. The study's exempt status was confirmed by the IRB Ile-de-France. During the study period, women were routinely informed that their records could be used for evaluation of medical practices and that they had the right to opt-out of these studies. This was a retrospective analysis of a series of severe-stage DIE patients without a prior history of surgery for endometriosis and who had a monofetal pregnancy followed in a tertiary care center. Women with a diagnosis of DIE based on MRI criteria [5, 33] obtained before pregnancy were retained for the study. After delivery, an MRI was carried out of women with DIE due to painful symptoms.

### Patients

All of the pregnant women received prenatal care at our tertiary university hospital before 22+0 weeks and they gave birth there after 24+0 weeks in accordance with the protocols of the department.[34]

### Imaging protocol

The two sets of pelvic MRI examinations were performed by a single experienced radiologist, who is referring practitioner for image-based diagnosis of endometriosis, using the same protocol before and after pregnancy on a 1.5 T MRI machine (Sonata, Siemens; Erlangen, Germany) based on stringent previously published criteria.[5, 7, 8, 35, 36] The MRI obtained before and after pregnancy were reviewed independently, one month apart, by the same experienced radiologist to avoid memory contamination and inter-operator variability.[37] In keeping with the literature, OMA exhibited pathognomic features of signal loss on T2-weighted imaging, referred to as “T2 shading”, and high signal intensity on T1-weighted images with and without fat saturation. These elements reflect the hemorrhagic nature of OMA and they help differentiate them from other T1-hyperintense lesions, such as dermoid and hemorrhagic cysts.[38, 39] DIE nodules are composed of fibromuscular hyperplasia surrounding endometrial glands, displaying T2-hypointense signal, T1 intermediate signal intensity, with masses that had irregular edges and/or thickening of soft tissues leading to distortion of the normal pelvic anatomy and adhesion formation.[38, 40] Deep endometriosis involving the uterosacral ligaments appears as T2-hypointense thickening or nodularity. Rectosigmoid involvement exhibits a specific morphological pattern, in the anterior wall, featuring a semi-lunar hypointense T2 signal nodule, recently described as a “mushroom cap sign”.[41] The DIE lesions were classified based on five locations (i.e., uterosacral ligament(s), the vagina, bladder, intestine, and ureter),[42] and they were diagnosed according to stringent imaging criteria published elsewhere.[36] In cases with multiple DIE lesions, the patients were classified according to the worst location (least to most severe: uterosacral ligament(s), the vagina, bladder, intestine, and ureter).[42] The volumes of the DIE lesions and the sizes of the OMA were assessed by MRI before and after the pregnancy by the same radiologist (AEM). The nodule volume of the uterosacral ligament(s), the bladder, and the intestine were calculated with the volume formula for a general ellipsoid solid (V = π/6 [(a + b)/2]). Posterior vaginal fornix nodules were considered to be spheroids, and the volumes were calculated with the volume formula for a general sphere (V = 4/3 π r^3^).[43]

### Data analysis

For each patient, their personal history data and pregnancy-related information were collected including the maternal age, weight, size, assisted reproductive technology (ART), gestity, parity, gestational hypertension, mode of delivery, gestational age at delivery, preterm birth < 37 weeks of gestation, birth weight, small for gestational age offspring, the time interval between the MRI before pregnancy and delivery, and the time interval between delivery and the MRI after pregnancy. For each patient, the distribution of DIE lesions and the presence of OMA were recorded.

### Statistical analysis

The continuous data are presented as means with the standard deviation and as medians and the interquartile range (IQR) 25–75%. Paired analyses were used when comparing pre- and post-pregnancy lesion volumes (mm^3^) by MRI, and comparisons were performed according to the DIE location. In case of multiple DIE lesions, paired comparisons of lesion volumes before and after pregnancy were also performed according to the largest lesion of each patient. Paired OMA sizes (mm) were compared before and after pregnancy. The quantitative data were compared using the Wilcoxon matched-pairs test. A p-value < 0.05 was considered statistically significant. The data were analyzed using STATA software for Macintosh (Stata/IC 11.0 for Mac, StataCorp College Station, TX, USA).

## Results

Between October 2012 and December 2016, 21 DIE patients without a prior history of surgery for endometriosis underwent MRI before and after pregnancy. The characteristics of the women and the pregnancies are presented in Table 1. No endometriosis-related complications were reported during the pregnancies. All of these 21 women were undergoing hormonal treatment at the time of the MRI prior to the pregnancy. The mean time interval between the MRI before pregnancy and delivery was 19.6 ± 8.5 months (median: 17.6, IQR 13.5–25.2 months). The mean time interval between delivery and the MRI after pregnancy was 11.0 ± 6.4 months (median: 8.3, IQR 6–15.2 months). The mean number of DIE lesions was 3.2 ± 1.1 (median: 4, IQR 3–4) (Table 1). The distribution of the DIE location and the OMA based on MRI are presented in Table 2: 19/21 patients exhibited vaginal posterior fornix involvement (90.5%), 20/21 uterosacral involvement (95.2%), 14/21 bowel involvement (66.7%), 3/21 bladder involvement (14.3%), and 15/21 OMAs (71.4%) (Table 2).

**Table 1:** Characteristics of the women and the pregnancies.

Items	n = 21
Maternal age, years ± SD (Min Max)	33.7 ± 3.2 (26.9; 39.1)
Height, cm ± SD	166.3 ± 6.3
Weight, kg ± SD	58.9 ± 6.2
ART, n;(%)	9 (42.8)
Gestity, mean ± SD	1.1 ± 0.7
Parity, mean ± SD	0.2 ± 0.4
Time interval between the MRI before pregnancy and the delivery, m (Min; Max)	19.6 ± 8.5 (5.2; 39.5)
Time interval between the delivery and the MRI after pregnancy, m (Min; Max)	11.0 ± 6.4 (3.1; 22.9)
C-section, n (%)	6 (28.5)
Number of DIE lesions, mean ± SD	3.2 ± 1.1
Hypertensive disease, n (%)	1 (4.8)
Gestational age at delivery, WG	38.4 ± 2.9
Preterm birth < 37 WG, n (%)	3 (14.3)
SGA, n (%)	1 (4.8)

ART: assisted reproductive technology; WG: weeks of gestation, SGA: small for gestational age offspring.

**Table 2:** Distribution of the endometriotic lesions.

Patient	Posterior vaginal fornix	Uterosacral Ligaments*	Bowel	Bladder	OMA*
Case 1	1	0	0	0	1
Case 2	1	2	0	0	1
Case 3	1	2	1	0	2
Case 4	1	1	0	0	0
Case 5	1	2	1	0	0
Case 6	1	1	1	1	1
Case 7	1	1	1	0	1
Case 8	1	1	1	0	1
Case 9	1	1	1	1	0
Case 10	1	1	1	0	1
Case 11	0	1	0	0	1
Case 12	1	2	1	0	0
Case 13	1	1	1	1	1
Case 14	0	1	0	0	0
Case 15	1	2	1	0	2
Case 16	1	2	1	0	2
Case 17	1	2	0	0	1
Case 18	1	2	1	0	2
Case 19	1	2	1	0	1
Case 20	1	2	0	0	0
Case 21	1	2	1	0	1

OMA: Ovarian endometrioma

* 1: unilateral lesion 2: bilateral lesion

**Fig 1:**
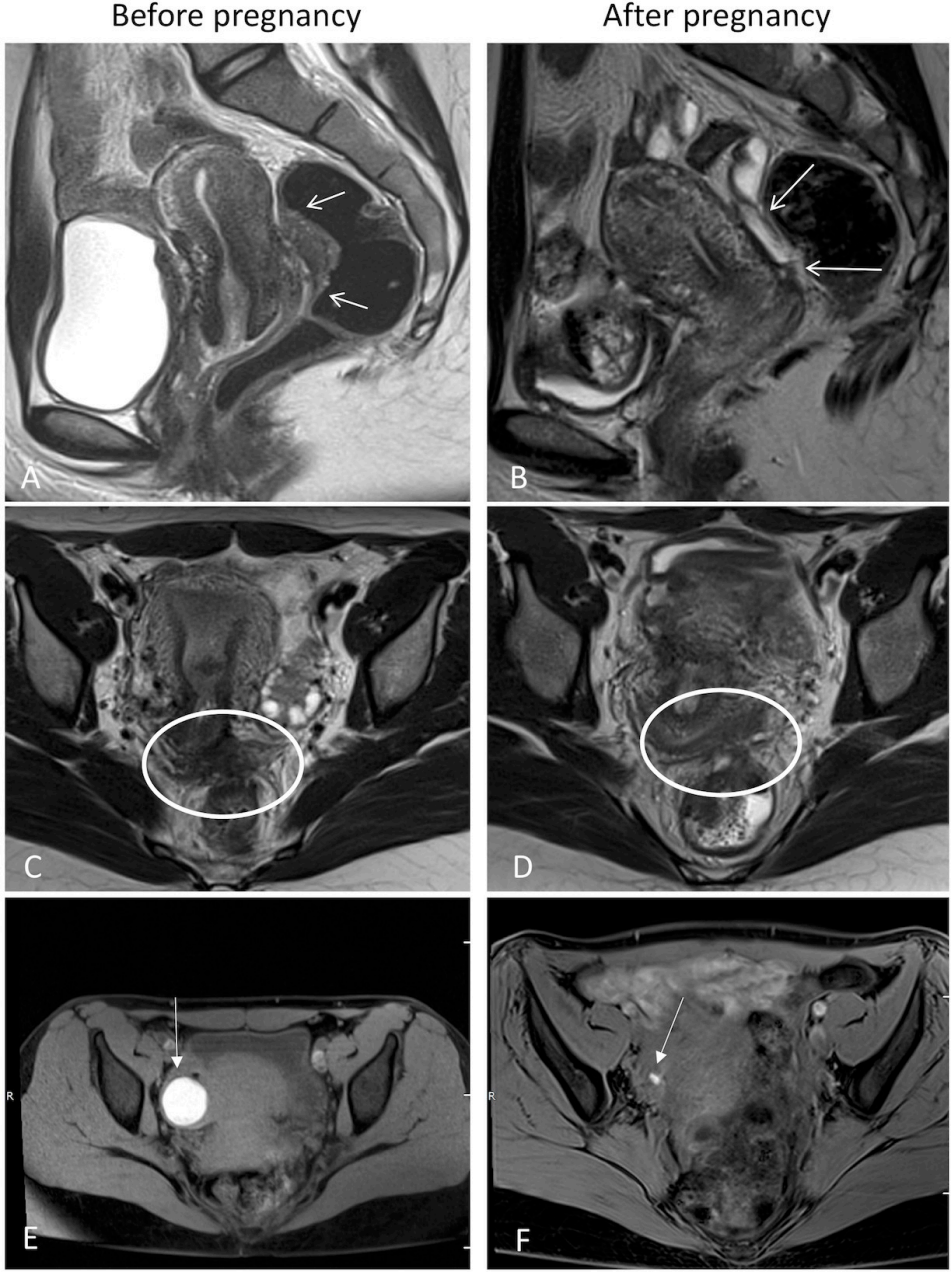
Magnetic resonance imaging of endometriosis, before and after pregnancy. (A,B) sagittal plane, deep infiltrative endometriosis of the bowel (white arrow); (C,D) axial plane, deep infiltrative endometriosis of the uterosacral ligaments and posterior vaginal fornix (white circle); (E,F) axial plane, ovarian endometriosis, axial plane with fat saturation, illustrating a left endometrioma in T1-hypersignal (white arrow).

Fig 1 depicts the DIE lesions of a woman before (Fig 1A, 1C and 1E) and after (Fig 1B, 1D and 1F) pregnancy based on MRI, with the bowel (Fig 1A and 1B), uterosacral ligaments and the posterior vaginal fornix (Fig 1C and 1D), and OMAs (Fig 1E and 1F) exhibiting a qualitative thinning and reduction in the size of the lesions.

A total of 67 DIE lesions were compared before and after pregnancy. For these DIE lesions, the volume decreased for n = 49/67 lesions (73.1%), was stable for n = 5/67 lesions (7.5%), and increased for n = 13/67 lesions (19.4%). The mean overall DIE lesion volume by MRI was significantly higher before pregnancy compared to after pregnancy (2,552 ± 315 mm^3^ vs. 1,708 ± 3,266 mm^3^, respectively, p < 0.01) (Table 3). In case of multiple DIE lesions, the mean volume of the largest lesion of each patient was significantly higher before pregnancy compared to after pregnancy (4,728 ± 4,776 mm^3^ vs. 3,165 ± 5,299 mm^3^; p < 0.01) (Table 3). The OMA sizes were significantly higher before pregnancy (Table 3).

**Table 3:** Comparison of the volumes of the DIE lesions and the sizes of the OMAs before and after pregnancy by MRI.

Mean DIE lesion volume by MRI	n	Before pregnancy	After Pregnancy	P value
Overall DIE lesions, mm^3^	67	2,552 ± 3,315	1,708 ± 3,266	< 0.01
Largest DIE lesion per patient, mm^3^		4,728 ± 4,776	3,165 ± 5,299	< 0.01
USL lesions, mm^3^	31	1,411 ± 1,485	518 ± 480	< 0.01
Posterior vaginal fornix, mm^3^	19	2,741 ± 3,456	2,577 ± 3,318	0.57
Bowel lesions, mm^3^	14	4,245 ± 4,579	3,342 ± 5,552	0.06
Bladder lesions, mm^3^	3	5,245 ± 5,865	873 ± 553	0.50
**Mean OMA size by MRI** OMA, mm	19	11.3 ± 10.0	6.4 ± 6.6	0.03

DIE: deep infiltrating endometriosis, OMA: endometrioma, MRI: magnetic resonance imaging, USL uterosacral ligament. All of the measurements are presented as means ± the standard derivation.

## Discussion

This study provides the first report of MRI-based DIE lesion size changes before and after pregnancy. We found that there was a significant decrease in the volume of the DIE lesions after pregnancy. The size of the OMA was also significantly reduced.

The strength of this study lies in the following aspects of the methodological design: (i) the MRIs, before and after pregnancy, were performed on the same patient: each patient was hence their own control; (ii) the MRIs were performed in a referral center by an experienced radiologist who is a referring practitioner for image-based diagnosis of endometriosis. It is now well accepted that, with experienced radiologists, imaging work-up allows for a non-surgical diagnosis of endometriosis;[5, 6] (iii) there had to be a one-month delay between the two MRI readings (before and after pregnancy) for each woman in order to limit any memory bias;[44] (iv) none of the women had a history of surgery for endometriosis, given the potential impact of pregnancy on DIE lesion progression.

The main shortcoming of our study stems from the lack of information regarding the hormonal cycle and lactation status of the women after delivery when the post-partum MRI was performed. Endometriosis lesions have not been shown to be the result of cellular growth, as is the case for tumors.[45, 46] Instead, endometriotic lesions undergo progressive fibrogenesis, starting with repetitive ectopic endometrial deposits that further evolve into fibrosis and smooth muscle metaplasia.[47, 48] DIE lesions should not be significantly influenced by fluctuations in extrinsic hormonal levels, which are very similar in women without endometriosis.[49] Yet, the local estrogen levels within lesions are significantly elevated [50] and endometriotic stromal and epithelial cells can be affected by menstrual phases, but not the extent of lesional fibrosis. Nevertheless, there have been reports of medical treatment of bladder or bowel DIE using low-dose oral contraceptives, aromatase inhibitors, GnRH agonists or dienogest,[51, 52] with various efficacies. In addition, it is unclear whether lactation has an impact on endometriosis lesions as there have been few studies to date in this regard.[11]

Our study highlights that there appears to be a significant decrease in the volume of DIE lesions after pregnancy. Of note, 13/67 (19.4%) of the DIE lesions increased in volume. A recent report in the literature that investigated a small number of cases of DIE lesions during pregnancy by ultrasound demonstrated a significant reduction in the size of DIE plaques, and that the nodules appeared to be less fibrotic and more homogeneous, with ill-defined contours.[30] Our data also highlight a significant reduction in the size of OMAs. There was no persistent decidualization after pregnancy among the 19 reported cases of OMA, which is similar to what has been reported elsewhere.[27, 53, 54] Although there is limited data regarding the growth dynamics of OMA with pregnancy, most investigators have reported regression or cessation of growth during pregnancy, thus corroborating our data.[53, 55] In a retrospective study, Ueda et al. reported on the natural progression of 25 ovarian endometriotic lesions observed during pregnancy in 24 women, with a decrease in the volume for 13 lesions (52%), and an increase in the volume for 5 lesions (20%).[32]

Our results support the hypothesis that a specific hormonal environment during pregnancy may positively impact the appearance of DIE lesions through several processes: (i) amenorrhea and the absence of cyclic retrograde fallopian bleeding limiting peritoneal and ectopic implant stimulation;[3] (ii) anovulation secondary to the well-known hormonal ovarian blockade occurring during pregnancy;[56] (iii) high placental production of steroid hormones that may directly modify endometriotic lesions,[57], with a decrease in the intra- and peri-lesional inflammatory status and reduced production of prostaglandins and cytokines;[13] (iv) decidualization of the endometrium during pregnancy, corresponding with the transformation of stromal fibroblasts into epithelioid-like decidual cells and an adjoined massive influx of immune cells,[26] may participate in intrinsic changes that could persist after delivery.

In terms of clinical aspects, previously published studies have reported a beneficial role of pregnancy on endometriosis-related symptomatology,[30, 58–60] without any data regarding the change in the volume and the size of endometriotic lesions based on MRI. Our present study supports the hypothesis of a favorable effect of pregnancy on the volume of DIE lesions. In clinical practice, these results raise the question of the need to systematically perform surgery before pregnancy for DIE patients.[33] However, it is important to stress that, although rare, severe cases of complications during pregnancy, including spontaneous hemoperitoneum,[61] digestive perforation,[62] and ureteral rupture [63] have been linked with the puerperal changes of ectopic lesions. Further studies are necessary to more precisely define the indications for surgery for DIE patients who wish to become pregnant, since there are is no clear evidence that surgery can prevent obstetrical risk in case of endometriosis.[64]

## Conclusion

This study demonstrates, for the first time, a favorable effect of pregnancy on the volume of DIE lesions, which regressed significantly after pregnancy based on MRI evaluation. Longer follow-up is required to more extensively evaluate the change in DIE lesions during and after pregnancy.

## Supporting information

S1 Dataset Clinical and anatomical data. (XLSX)
